# The effect of formal, neonatal communication-intervention training on mothers in kangaroo care

**DOI:** 10.4102/phcfm.v6i1.675

**Published:** 2014-11-06

**Authors:** Alta Kritzinger, Elise van Rooyen

**Affiliations:** 1 Clinic for High-Risk Babies (CHRIB), Department of Speech-Language Pathology and Audiology, University of Pretoria, South Africa; 2 Kangaroo Mother Care Unit, Department of Paediatrics, Kalafong Hospital, University of Pretoria, South Africa

## Abstract

**Background:**

Due to low-birth-weight, preterm birth, HIV and/or AIDS and poverty-related factors, South Africa presents with an increased prevalence of infants at risk of language delay. A Kangaroo Mother Care (KMC) unit offers unique opportunities for training.

**Aim:**

The aim of the present study was to determine if formal, neonatal communication-intervention training had an effect on mothers’ knowledge and communication interaction with their high-risk infants.

**Methods:**

Three groups of mothers participated: Group 1 was trained whilst practicing KMC; Group 2 was not trained but practiced KMC; and Group 3 was also not trained but practiced sporadic KMC. Ten mothers per group were matched for age, education level and birth order of their infants. The individual training was based on graded sensory stimulation and responsive mother-infant communication interaction, which emphasised talking and singing by the mother.

**Results:**

Significant differences were found in mother-infant communication interaction between all three groups, which indicated a positive effect on Group 1 with training. Group 2, KMC without training, also had a positive effect on interaction. However, Group 1 mothers with training demonstrated better knowledge of their infants and were more responsive during interaction than the other two groups.

**Conclusion:**

The present study suggests that neonatal communication-intervention training adds value to a KMC programme.

## Abstrait

**Les effets de la formation formelle d'intervention et de communication néonatales chez les mères ayant recours à la méthode kangourou**.

**Contexte:**

En raison d'un poids de naissance trop faible, de la prématurité, de facteurs tels que le VIH et/ou le SIDA et de la pauvreté, les risques de retard de langage chez les enfants sont plus prévalents en Afrique du Sud. Le service des soins maternels de type kangourou (KMC) offre une opportunité unique de formation.

**Objectif:**

L'objet de cette étude est de déterminer si la formation formelle d'intervention et de communication néonatales avait un effet sur le savoir et l'interaction communicative des mères avec leurs enfants à haut risque.

**Méthodes:**

Trois groupes de mères y ont participé: le Groupe 1 a été entrainé en pratiquant le KMC; le Groupe 2 n'a pas été entrainé mais a pratiqué le KMC; et le Groupe 3 n'a pas non plus été entrainé mais a pratiqué sporadiquement le KMC. On a réuni dix mères par groupe selon l’âge, le niveau d’éducation et l'ordre de naissance de leurs enfants. On a basé l'entrainement individuel sur la stimulation sensorielle et l'interaction communicative mère-enfant, où la mère parle et chante à l'enfant.

**Résultats:**

On a remarqué des différences importantes dans l'interaction communicative mère-enfant parmi les trois groupes, ce qui a montré un effet positif sur le Groupe 1 entrainé. Le Groupe 2, avec le KMC sans entrainement, a aussi eu un effet positif sur l'interaction. Cependant, les mères du Groupe 1 entrainées ont fait preuve d'une meilleure connaissance de leur enfant et réagissaient mieux au cours de l'interaction que les deux autres groupes.

**Conclusion:**

Cette étude montre que la formation d'intervention communicative néonatale ajoute de la valeur au programme KMC.

## Introduction

Kangaroo mother care (KMC) has a strong impact and focus on mother-infant communication interaction.^1, 2^ Therefore, speech-language therapists should support and utilise this method to develop neonatal communication-intervention programmes for high-risk infants and their caregivers. In KMC, a neonate is securely carried in an upright position, skin-to-skin, between the mother's breasts. This evolutionary, reclaimed care pattern offers benefits to the infant, the parents and family, healthcare systems, and to speech-language therapy practice.

Kangaroo mother care stabilises a neonate's physiological functions,^1^ improves behavioural and state of alertness regulation and may enhance brain organisation and neuromaturation in preterm infants, as sleep patterns are better organised and cyclical.^3^ Cyclical sleep during the foetal period, and when interrupted by preterm birth, is important for early sensory development.^4, 5^ The benefits of KMC to the mother include: increased oxytocin levels in herself and her infant, resulting in enhanced lactation and bonding to her new-born infant; and reduced stress, anxiety and postnatal depression.^1^ Family members, especially fathers and grandmothers, can also participate in kangaroo care.^1^ Since the neonate is discharged earlier from hospital than with conventional incubator care,^6^ and the mother is already skilled in caring for the very small baby at home, the family rather than the hospital is central to the infant's caregiving. Research indicates that mothers and families from different cultures worldwide respond spontaneously and favourably to KMC when trained to apply the procedure.^7^ Although the physical implementation of KMC is only applicable during the first weeks or months of an infant's life, the benefits of the intervention are far reaching.

All the benefits of KMC converge at a point where the ideal opportunity arises for speech-language therapists to intervene early and ameliorate threats to typical language development in high-risk infants. Recent studies have clearly shown that those children born preterm, even those born late preterm, are at risk of language and academic difficulties.^8^ As described by ASHA,^9^ KMC endorses some of the most important principles of early communication-intervention. Similar to early-intervention principles, KMC: is family centred, culturally responsive and developmentally supportive; represents a natural environment of care and prolonged breastfeeding; is team based; starts as early as possible in a child's life; and is supported by high-quality evidence.

**FIGURE 1:**
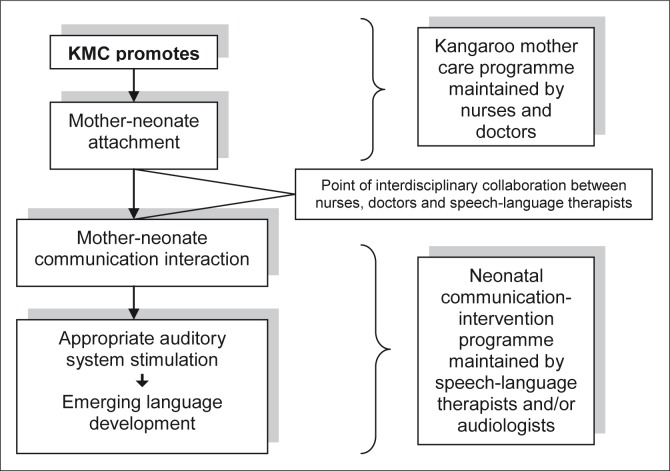
The interdisciplinary relationship between a KMC programme and a neonatal communication-intervention programme. KMC, Kangaroo Mother Care.

There is also an additional rationale as to why speech-language therapists should be involved in a KMC unit or a neonatal intensive care unit (NICU) where the intervention procedure is promoted. When mothers and their high-risk neonates are discharged from a KMC unit they disperse to areas where follow-up early intervention services may not be accessible to them. Since the average lodging of a mother and infant in the KMC unit, where the research was conducted, is 13 days,^6^ the unit offers a short period of unique access to mothers and their high-risk neonates. The availability of mothers, who already have an increased interest in their infants as they have been primed by KMC, provides a valuable opportunity for speech-language therapists to start an interdisciplinary communication-intervention programme in a KMC unit (see Figure 1). Nurses and doctors train mothers to practice KMC, whilst speech-language therapists can train them at the same time to appropriately facilitate their infants’ communication development. The continuous practice of KMC over time allows for strong bonds to develop between mother and neonate.^10^ Mother-infant communication interaction results naturally from attachment, and, as stated by Billleaud,^11^ attachment forms the basis of mother-infant communication interaction (see Figure 1). Whilst the mother is gradually becoming familiar with her infant through continuous KMC, she also needs information to understand her preterm infant's progress through developmental stages, as described by Gorski, Davidson and Brazelton,^12^ and Rossetti.^2^. The mother needs to identify the infant's stress signs, its readiness for stimulation, and its subtle cues to communicate. A mother may require training to establish a highly sensitive pattern of successful reciprocal communication interaction with her preterm infant^2^ so that the child's auditory system is appropriately stimulated to facilitate language development during the replaced foetal period.

The importance of appropriate auditory system stimulation in preterm infants is recognised in the literature. According to Graven and Browne,^4^ the period from 25 weeks gestation to 5–6 months postnatal age is most sensitive to the neurosensory development of the auditory system. The developing auditory system requires stimulation by the human voice, language and music during the last 10–12 weeks of foetal life so that the hair cells of the cochlea, the axons of the auditory nerve and the neurons of the auditory cortex are tuned to receive specific frequencies and intensities – a function that can only develop when ambient noise is not louder than 60 dB.^4^ The foetus appears to receive an ideal signal-to-noise ratio of auditory input in the womb. The effects of noise exposure on preterm infants were demonstrated by evidence of delayed auditory cortex organisation found in mouse pups when reared in the continuous presence of white noise.^13^ Considering the unique potential of the early human auditory system to make language acquisition possible, there appears to be a balance between stimulation provided by the ambient noise of the maternal heartbeat and blood circulation, other internal and external noises, and the consistency of a single maternal voice heard *in utero*. The ideal stimulation of the foetal auditory system during the third trimester of pregnancy, provided by the consistency of the mother's voice against vastly different and diffuse background noises, is interrupted by preterm birth. Logic follows that an appropriate ratio of signal-to-noise auditory compensation must be provided in the caregiving environment during the infant's unplanned perinatal period.

Since the full-term infant is born with a preference for the maternal voice^14^ it is clear that complex auditory processing starts before birth and that antenatal auditory system stimulation is required. It is hypothesised that the exclusive maternal-caregiving environment created by continuous KMC provides some form of compensatory auditory system stimulation and possibly an appropriate early language-learning environment for preterm infants (see Figure 1). In the upright KMC position, the infant's ears are in close approximation to the mother's mouth so as to hear her voice. The ambient noise, in contrast to a NICU with much noise created by staff and lifesaving equipment, is typically subdued in a dedicated KMC unit. If a mother is taught to be sensitive to the infant's stress signals, she may provide the appropriate amount and quality of auditory stimulation according to her preterm baby's developmental stage, as described by Gorski et al.^12^ and Rossetti.^2^

A further justification to implement a neonatal communication-intervention programme is the high prevalence of at-risk infants born in South Africa, mostly due to low birth weight, preterm birth, HIV and/or AIDS and persistent poverty in many families. If the opportunity to start intervention in the neonatal period is missed in South Africa, case finding may be complicated by a lack of early-intervention services at primary and secondary healthcare facilities. Primary healthcare workers have little capacity to identify infants that require early intervention due to their workload, staff shortages and lack of training.

Since the establishment of the KMC unit at the hospital where the study was conducted, the parent-education programme provided by nurses and doctors has included training and support regarding the KMC method, breastfeeding, and the caring for low-birth-weight infants in the unit and after discharge.^6^ As the KMC programme provided limited education to the mothers to enhance the infants’ communication development, the opportunity was used to develop a formal communication-based training programme for the mothers, as indicated in Figure 1. The need to contribute to evidence-based neonatal intervention programmes in South Africa provided the stimulus for the research study.

## Aim and objectives

The objective of the study was to determine if the short-term knowledge and communication interaction of mothers with their high-risk neonates improved with formal training. To achieve the objective, the participants’ language proficiency and confidence had to be determined first in order to establish their readiness for training in English.

## Research methods and design

A three-group comparison design was selected to determine whether an experimental group of mothers that received formal communication-intervention training behaved differently with training, as opposed to the two groups who received no training.^15^. Group 1 was trained whilst practicing continuous KMC in a well-established KMC unit; Group 2 was not trained, but practiced KMC in the same unit; and Group 3 was also not trained but practiced sporadic KMC at another hospital. The Group 2 was selected after all the mothers of the first group were discharged from the KMC unit. As the KMC unit consisted of one large dormitory, each group's data were collected consecutively to prevent the knowledge of the trained group to influence the first control group, who practiced KMC but did not receive training. The research method is outlined in Table 1.

**TABLE 1:** Research design and methods.

Participant groups	Without training	Neonatal communication-interaction training	Post training measurements after two weeks
Kalafong Hospital Continuous KMC	Interview	3 training sessions over two weeks	- Interview - Video recording
Kalafong Hospital Continuous KMC	None	No training	- Interview - Video recording
Another hospital Sporadic KMC	None	No training	- Interview - Video recording

KMC, Kangaroo Mother Care.

As seen in Table 1, the interview with the individual mothers was conducted as a pre-test/post-test to determine whether qualitative differences in knowledge of their infants occurred in Group 1 without and with training. Multiple sources of data were utilised to support the mixed methods design of the study. Data were collected from case histories in medical files (quantitative data), interviews with the mothers (text data) and video recordings of mother-infant communication interaction, analysed according to a rating scale.

### Ethical considerations

The Research Ethics Committee of the Faculty of Humanities and the Faculty of Health Sciences (Protocol number 40/2004), University of Pretoria and the Provincial Department of Health gave permission to conduct the study. A research assistant informed prospective participants, in English or Setswana, about the aims and procedures of the study. Participant information brochures were available in English and Setswana, the home language of most of the participants. All participants gave written informed consent to participate in the study and to be video recorded. The infants were in stable conditions when their mothers handled them, and when they were video recorded. The researcher handled none of the infants. After the study, a copy of the video recording was given as an incentive to each participant.

### Participants and setting

A total of 30 participants, forming three groups of ten mothers in each group, were utilised in the study. The two hospitals were tertiary-level public facilities that received patients from surrounding townships, the inner city or rural towns. The participants of Groups 1 and 2 lodged with their infants in the KMC unit of the first hospital, whilst the participants of Group 3 did not stay in the hospital and had to come in from home to visit their infants in the neonatal unit.

The three groups were matched according to mothers’ ages, level of education (either primary or secondary school education), and birth order of their infants. The groups were very similar in composition of factors that could relate to experience of motherhood. Prior experience in motherhood, as measured by the number of children of a participant, was an important variable to equalise between the groups, as parental experience could have given participants an unfair advantage over those with no previous parenting experience.

As further indicated in Table 2, the groups differed in the infants’ birth weights, age at the time of the video recordings, number of days in the KMC unit or the neonatal unit, and gender (See Table 2, numbers 4 to 7). The variability of the sample may be a reflection of the diversity that can be expected in the high-risk neonatal population. The differences between the three groups were considered when the results were discussed.

**TABLE 2:** Description of participants (*n* = 30).

Characteristics of mothers and infants	Group 1 (*n* = 10) Continuous KMC and training*	Group 2 (*n* = 10) Continuous KMC, no training	Group 3 (*n* = 10) Sporadic KMC, no training
**Dependent variables similar across the three groups**			
Mother's age *p*-value: 0.5507	Mean: 27.5 years s.d.: 5.3 Minimum: 20 years Maximum: 36 years	Mean: 29 years s.d.: 6.4 Minimum: 19 years Maximum: 38 years	Mean: 30.3 years s.d.: 5.5 Minimum: 23 years Maximum: 40 years
Educational level	Primary school: 2 Secondary school: 8	Primary school: 2 Secondary school: 8	Primary school: 2 Secondary school: 8
Gravida (number of children, current infant included) *p*-value: 0.9307	Mean: 1.9 children s.d.: 0.7 Minimum: 1 Maximum: 3	Mean: 1.9 children s.d.: 0.8 Minimum: 1 Maximum: 4	Mean: 2.5 children s.d.: 1.9 Minimum: 1 Maximum: 6
**Dependent variables not similar across the three groups**			
Birth weight of infants (no *p*-values obtained)	Mean: 1613 g Minimum: 1000 g Maximum: 2300 g	Mean: 1335 g Minimum: 978 g Maximum: 1780 g	Mean: 1351 g Minimum: 880g Maximum: 2610 g
Age of infants at video recording *p*-value: 0146**	Mean: 29.5 days s.d.: 13.9 Minimum: 16 days Maximum: 33 days	Mean: 23.5 days s.d.: 8.5 Minimum: 9 days Maximum: 33 days	Mean: 13.6 days s.d.: 13.53 Minimum: 3 days Maximum: 47 days
Number of days in KMC *p*-value: 0.0063**	Mean: 18.4 days s.d.: 10.3 Minimum: 9 days Maximum: 42 days	Mean: 8.8 days s.d.: 3.19 Minimum: 4 days Maximum: 13 days	Mean: 13.6 days in neonatal unit s.d.: 13.5 Minimum: 3 days Maximum: 47 days
Gender of infants	Boys: 9 Girls: 1	Boys: 4 Girls: 6	Boys: 4 Girls: 6

KMC, Kangaroo Mother Care. Training*, Neonatal communication-interaction training. p**, represent significant differences between the three groups on the 0.05% level

### Participant selection

Participants were purposely selected to participate in the study. Criteria for selection included that participants had to be mothers of preterm (born before 37 weeks gestation) and low-birth-weight infants (birth weight < 2 500 g), either in a KMC programme (Groups 1 and 2) or in a neonatal unit (Group 3). Mothers of infants who received oxygen at the time of data collection, infants with congenital disorders, and twins were excluded from the study since these variables were considered to be stressors that could have influenced the mother-infant interaction negatively.

A participant language proficiency and confidence rating scale was designed to rate the participants’ language proficiency and perceived confidence, respectively. This was done in order to ensure that the participants were not unfairly judged on their English language skills or intimidated by the researcher. The aim was to recruit participants who scored higher than two points on the rating scale. The 4-point rating scale was used by the research assistant and researcher whilst recruiting the participants, and is presented in Table 3. The research assistant and researcher held informal conversations with prospective participants in English. The research assistant and researcher completed the rating scale separately. A consensus discussion was held when there were differences in the ratings. Mothers who scored lower than two were not included in the study, as they would not have communicated successfully in English.

**TABLE 3:** Participant language proficiency and confidence rating scale.

Rating	English language proficiency	Perceived confidence
1	Participant did not understand all questions.	Participant was quiet and did not make eye contact with researcher.
2	Participant understood all questions after rephrasing.	Participant answered questions, but did not volunteer information.
3	Participant understood all questions, but did not elaborate on her answers.	Participant made eye contact and answered all questions.
4	Participant answered questions in full sentences.	Participant initiated conversation with researcher, volunteered information.

Group 1 was selected first. Ten participants received three individual training sessions over 14 days whilst they were practicing KMC. A short interview was conducted with them, before and after their training (see Table 1). Group 2 was selected after all the mothers of the first group had been discharged from the KMC unit. The waiting period was necessary so that Group 1 could not model the trained communication-interaction behaviours with their infants to the group who did not receive training. The participants in Group 2 received no training, but were practicing continuous KMC with their infants in the unit. Group 3 neither practiced continuous KMC, nor did they receive any training to facilitate communication interaction with their infants. As KMC is now widely practiced in South Africa,^16^ no state hospital in the area could be found that did not practice KMC at all. The hospital where Group 3 was recruited did not have a KMC unit at the time of data collection, but encouraged mothers to practice KMC when they visited their infants in the neonatal unit. Some mothers in Group 3, therefore, practiced sporadic KMC but did not lodge with their infants in the hospital.

### Material and apparatus

Three sets of instruments were used to collect data. The first instrument was a self-designed checklist to record the demographic and biological characteristics of the participants and their infants from the medical files. The second was a self-designed interview schedule to determine participants’ knowledge of their infants’ communication abilities and development. Five main questions with follow-up questions were posed to the participants during the individual interviews:

1. Do you think your baby can hear?
  - If the response was yes, the following question was asked:
    - How do you know?
  - If no, the following question was asked:
    - When do you think your baby will be able to hear?
2. Do you think your baby can see?
  - If the response was yes, the following question was asked:
    - How do you know?
  - If no, the following question was asked:
    - When do you think your baby will be able to see?
3. Do you know when your baby is tired/needs a break?
  - If yes, the following question was asked:
    - How do you know?
4. Do you know when your baby is happy?
  - If yes, the following question was asked:
    - How do you know?
5. What else can your baby do?

Third, a 4-point scale from Klein and Briggs^17^ was used to rate the participants’ communication interaction with their infants (see Table 6 later). The participants’ interaction with their infants were video recorded and rated as: (1) rarely or never; (2) sometimes; (3) often; and (4) optimally occurring according to the *Observation of Communication Interaction*.^17^ The apparatus included a portable Panasonic VHS-C NV-R33 movie camera, with a hi-fi stereo microphone, a 10 x wide lens and an image stabiliser.

**TABLE 4:** Results of participant language proficiency and perceived confidence.

Variables	Group 1	Group 2	Group 3
Language proficiency of participants in averages (maximum 4).	2.7	3.4	2.6
Perceived confidence of participants in averages (maximum 4).	3.0	3.4	2.9

The material also included the neonatal communication-intervention programme. The interdisciplinary parent-training programme is based on three components:

1. The practice of KMC, as explained by Van Rooyen et al.,^6^ is taught to the mothers by nurses and doctors. Tactile stimulation is provided to the infants by KMC (i.e. skin-to-skin touching) and vestibular stimulation occurs naturally when the mother is moving.
  The researcher, a speech-language therapist, taught the following two components:
2. Graded sensory stimulation^18^ is to be provided by the mother according to the three developmental stages of preterm infants: turning in, coming out and reciprocity stages.^12^, ^2^. When the infant is in the turning in and coming out stages, visual stimuli (attempts to make eye contact with the infant) are kept to a minimum. During these stages, the infant may meet attempts at eye contact with gaze aversion. Auditory stimulation is provided by the mother's own voice when she talks and sings softly to the infant during all three developmental stages. During the reciprocity stage, the visual stimulus of eye contact between mother and infant is gradually added. Singing and talking are maintained, provided that the infant is not over stimulated. As indicated by Graven and Browne,^4^, the importance of the mother's voice when she talks and sings to the infant is vital.
3. Responsive interaction between mother and infant^19^ is taught. The mother is trained to: identify stress signs in her infant; reduce the infant's stress by decreasing sensory stimuli, such as noise; hold the infant in the KMC position; only initiate interaction once the infant is regulated and calm. The mother is taught to respond to the infant's subtle cues for readiness to communicate and not to provide intrusive stimulation, as cautioned by Klein and Briggs.^17^ When the infant is in the reciprocal stage, face-to-face interaction is possible. When stress cues, such as gaze aversion, hiccupping, sneezing, couching, stretching arms and pushing feet are identified, stimulation is reduced and the infant is placed back in the KMC position.

### Procedures

A pilot study was conducted with three mothers to test the instruments and procedures, determine the participants’ level of understanding English and openness to exchange information with the researcher, who did not share the same culture. The pilot participants were found to be very willing to explain their infants’ progress in KMC and wanted new information. The main study proceeded as indicated in Table 1. Participants gave written informed consent. Participants had to be proficient in English to understand the training instructions and interview questions, as the researcher (also the trainer) was not proficient in Setswana. A video recording was made of the interviews and 10–15 min interaction time of the participants with their infants. The participants were asked to play with their infants or perform caregiving tasks whilst the video recording was running and the researcher was studying the medical file. At the beginning of the recording, the researcher assured each mother that her interaction with her infant was appropriate.

The individual communication-interaction training was conducted at each participant's bed by discussing and demonstrating desired behaviours. The researcher identified the infant's developmental stage according to the gestational age and chronological age, and explained it to the mother. Whilst interacting with the infant, the researcher pointed out the infant's hearing responses, eye contact and stress signs to the mother. The mother was gently guided to interact responsively with her infant, whilst encouraging her appropriate behaviours with verbal comments. The training started with the mother's focus of attention, which was usually her infant. The language barriers were managed by adapting vocabulary, following a flexible approach, keeping the conversation interactive, using minimal prompts to encourage the participants and asking open questions instead of questions requiring yes or no answers. With Group 1, after three training sessions over a period of approximately two weeks, a post-training video was recorded. The communication-intervention programme was given to each participant in the form of a handout. Participants of Group 2 and 3 were not trained, but received the handout after their video recordings were made.

### Data analysis

Standard deviations (s.d.) were calculated to indicate how far the values deviated from the mean. *P*-values were calculated by means of the Kruskall-Wallis one-way analysis of variance test or Fischer's exact test in order to determine if differences between the groups were statistically significant. The interviews were transcribed from the video recordings and the ‘yes’, ‘no’ and ‘I don't know’ responses were calculated out of ten. The most representative answer to open questions 1 – 4 was selected and reported in Table 5. All of the different themes identified in the answers to question 5 (*What else can your baby do?*) were presented in the results.

**TABLE 5:** Results of the interviews (*n* = 30).

Question	Group 1s Continuous KMC without training	Group 1 Continuous KMC with training	Group 2 Continuous KMC alone	Group 3 Sporadic KMC Neonatal unit
1. Do you think your baby can hear?	Yes: 10 Don't know: 0 No: 0	Yes: 10 Don't know: 0 No: 0	Yes: 9 Don't know: 1 No: 0	Yes: 6 Don't know: 2 No: 2
How do you know? Example	'Cannot say.'	'He turns his head when you talk.'	'When I touch him he becomes quiet.'	'He jerks if you bang the incubator.'
2. Do you think your baby can see?	Yes: 5 Don't know: 5 No: 0	Yes: 10 Don't know: 0 No: 0	Yes: 4 Don't know: 3 No: 3	Yes: 6 Don't know: 3 No: 1
How do you know? Example	'He sleeps.'	'He follows my hand if I move it.'	'Difficult to tell, she is so small.'	'She has eyes.'
If no, when will your baby be able to hear and see? Examples	'After one or two months.'	N/a	'Maybe after two weeks.'	'Maybe next month.'
3. Do you know when your baby is tired?	Yes: 5 No: 5	Yes: 10 No: 0	Yes: 7 No: 3	Yes: 4 No: 6
How do you know? Examples	'I can see when he is tired.'	'He shows the stop sign with his hand.'	'When he sleeps.'	'When he stretches.'
4. Do you know when your baby is happy?	Yes: 9 Don't know: 1 No: 0	Yes: 10 Don't know: 0 No: 0	Yes: 9 Don't know: 1 No: 0	Yes: 6 Don't know: 0 No: 4
How do you know? Examples	'He smiles.'	'He smiles when he is sleeping.'	'He is happy in KMC.'	'When he feeds.'
5. What else can your babydo? All themes of verbatim responses:	'Nothing.' 'Cries when hungry.' 'She likes moving.' 'Opens her eyes.' 'He looks, but he sees nothing.' 'He can sleep.' 'He likes to play.' 'Stretches.' 'He can suck now.'	'He drinks well.' 'He cries, sleeps.' 'Moves more.' 'Opens her eyes.' 'Cries for her food.' 'More responsive when she is awake.' 'She would stare at me for a long time.' 'She recognises my voice.' 'Drinks and stops, drinks and stops.' 'Sucks his thumb.' 'He can see better.' 'Sleeps at night, but (also) awake at night.' 'He follows me when I go away.' 'Smiles more.' 'Can do more things now.' 'Still sleeps too much.'	'Nothing.' 'Cries a lot.' 'Moves his legs and arms.' 'When I put him down he moves.' 'Pushes herself forwards.' 'She likes playing.' 'Responds when I talk to him.'	'Nothing.' 'Stretches his body.' 'Kicks.' 'I don't know' 'Moves.' 'Smiles.' 'Opens his eyes.' 'Cries sometimes.'

KMC, Kangaroo Mother Care.

### Reliability and validity

Evidence of the trustworthiness of the study, expressed as confirmability and internal validity, is as follows: each video recording was analysed three times by the researcher, and 33% of the recordings were analysed by a second rater, for whom the groups were concealed. The average of the ratings was calculated. Data were collected consecutively after Group 1 had been discharged, so that Groups 1 and 2 could not contaminate each other, as participants were selected from the same KMC unit. The objective was dependability, or a refined understanding of the setting. Language and cultural differences were recognised, strategies were applied to be culturally sensitive, to communicate effectively, and to allow trust and spontaneity to develop between the researcher and participants.

## Results

The first result describes the participants’ language proficiency and confidence, after which the differences in knowledge, mother-infant communication interaction and behaviour types with and without training will be presented.

### Participants’ language proficiency and confidence

As already determined by the Kruskall-Wallis one-way analysis of variance, the *p*-values indicate no statistical differences between the three groups, for the variables of age, educational level and the number of children (see Table 2). The equivalence between the groups was further determined by the participants’ English language proficiency and confidence, as perceived by the researcher and research assistant. A maximum of four points could be obtained. The results are presented in Table 4.

**TABLE 6:** Comparison of mother-infant communication interaction between the three groups (*n* = 30).

Test items adapted from Klein and Briggs^17^ (The mother …)	Group 1† (%)	Group 2 (%)	Group 3 (%)	*P*-values
1. Provides appropriate tactile and kinesthetic stimulation	90	60	30	0.0291
2. Displays pleasure whilst interacting with infant	100	90	30	0.0013
3. Responds to infant's distress	100	60	33.33	0.0482
4. Positions self and infant for eye-to-eye contact	90	40	20	0.0089
5. Smiles contingently at infant	80	28.57	0	0.0001
6. Varies prosodic features of speech when talking to infant	90	20	0	0.0001
7. Encourages conversation	80	30	0	0.0001
8. Responds contingently to infant's behaviour	80	40	20	0.0366
9. Modifies interaction in response to negative cues from infant	83.33	33.33	22.22	0.0985
10. Uses communication to teach language and concepts	30	0	0	0.0887

† Post training results.

When the decimals points in Table 4 are approximated, the numbers indicate an average value of three for all the groups. The ratings indicate that, on average, the participants understood all the questions asked by the researcher, but did not necessarily elaborate on the answers. The participants were sufficiently confident to answer all questions and made eye contact with the researcher (see rating 3, Table 3). Applying the confidence scale reminded the researcher to be culturally sensitive and to use appropriate interviewing skills when talking to the participants. The ratings of Group 2, who did not receive training, indicated a slight advantage over Groups 1 and 3.

### Knowledge of infants

According to Table 5, and based on the interviews, there was a clear difference in Group 1 before training and after two weeks of training. With training and whilst conducting KMC, Group 1 gave increased descriptions of their infants’ behaviours, supplied sophisticated descriptions by using terminology from the training programme (*he shows a stop sign with his hand*), demonstrated an understanding of neonatal listening and visual abilities, and knew about stress signs in their infants, as opposed to before the training.

Group 2 gave similar accounts of their infants to Group 1 without training, as both groups were conducting KMC without additional training at that stage. Without training, both groups were mostly sure that their infants could hear, were ambivalent about their infants’ vision and stress cues, and were mostly certain when their infants were content. Groups 1 and 2 practiced continuous KMC and gave better answers during the interview than Group 3. Participants in Group 3, who practiced sporadic KMC, could hardly describe their infants’ sensory abilities, cues and behaviours. The difference in knowledge of the infants between the two groups practicing continuous KMC, and the third group conducting KMC only sporadically, may indicate the positive effect of KMC on the first two groups with regards to understanding their infants’ behaviour. The fact that Groups 1 and 3 knew their infants longer than Group 2 (see Table 2, number 6), appeared not to have made a difference in the knowledge of their infants, which they displayed during the interviews. Group 1 and 2 were equally unsure about their infants’ abilities to hear and see, and whether their infants were tired or happy. Participants in Group 3 were least sure about these aspects of their infants’ behaviour.

### Mother-infant communication interaction

The results obtained through observation of mother-infant communication interaction, as presented in Table 6, show that all *p*-values indicate statistically significant differences between the three groups on the 0.05% level according to Fischer's exact test. Participants in Group 1 showed significantly higher scores on the mother-infant communication-interaction scale with training and KMC, as opposed to Groups 2 and 3 who did not receive training. The ten items of the communication-interaction scale included general interaction behaviours, assessing a mother's sensitivity to respond to her infant's immediate behaviour or withhold stimulation, and communication-specific behaviours. The general interaction behaviours included: holding and rocking (Item 1), displaying pleasure (Items 2 and 5), and responding contingently to the infant (Items 3, 8 and 9). The participants in Group 1 almost always positioned themselves so that the infant was able to make eye contact, talked ‘motherese’ to the infant by varying the pitch and tone of their voices, and encouraged conversation with the infant. Again, Group 1 and 2 showed better scores than Group 3. The age difference in the infants and the longer time spent with their infants in KMC could have advantaged Group 1 (see Table 2). Participants in Group 1 could have known their infants better than participants in Group 2 or 3. Participants in Group 3 did not know their infants well, as they had to come in from home to see their infants every day and did not spend sufficient time with their infants. Since the groups were matched for age, education level, gravida and birth weight, it may indicate that both KMC and the training programme had an effect on the participants’ communication interaction with their infants. None of the groups displayed optimal behaviours on Item 10, which may be too advanced for neonatal communication skills.

Further differences between the groups were observed. The different behaviours that the participants displayed whilst interacting with their infants were counted when the video recordings were analysed. The results are displayed in Table 7.

**TABLE 7:** Number of behaviour types displayed by participants whilst interacting with infants (*n* = 30).

Group	Mean	Median	s.d.	Minimum	Maximum
1	7.10 types	7	1.19	6	9
2	5.70 types	6	1.16	3	7
3	3.20 types	3	1.14	2	5

Note: *P*-value < 0.0001, indicating a significant difference between the 3 groups s.d, standard deviation.

### Behaviour types during mother-infant communication interaction

Statistically significant differences were found between the groups: Group 1 displayed the most behaviours, followed by Group 2, and then Group 3. The results confirm the findings of the mother-infant communication-interaction scale, where Group 1 mostly displayed optimal behaviours. The different types of behaviours displayed by the participants clearly show that their first interest was with their infants. A summary of the different caregiving behaviour types demonstrated by the three groups of participants is as follows: holds infant on her lap; touches infant's head, mouth, face, hands, feet; adjusts infant's position; puts infant down next to her; rocks infant; holds infant close, but not skin-to-skin; holds infant on her chest, skin-to-skin (KMC position); looks at infant; smiles at infant; kisses infant; wipes infant's mouth; changes nappy; feeds infant; talks to infant; strokes infant; covers infant with blanket; caresses infant; tickles infant; picks fluff from infant's blanket; adjusts blanket; adjusts infant's nasogastric tube; opens infant's blanket; pats infant gently.

As can be seen from these observations, the participants demonstrated a wide range of natural and spontaneous behaviours with their infants whilst the videos were recorded. It appeared that the participants were not inhibited by the unnatural communication-interaction context created by a video recording. The participants who talked to their infants demonstrated the typical features of ‘motherese’, as described by Gleitman, Newport and Gleitman,^20^ although it was not explicitly taught to them. One of the mothers in Group 1 softly sang a praise song, a traditional chant of ancestral names, to her infant.

## Discussion

On three different measures (interview, mother-infant communication interaction and types of behaviours), Group 1 (who practiced continuous KMC and received training in communication interaction with their infants) showed consistently better results with training. On average, the participants in Group 1 showed a high prevalence of communication-facilitating behaviours whilst interacting with their infants. It appears that, despite language and cultural differences between participants and trainer, the training had a direct, positive effect on the participants.

The significant differences on all measures between Group 2 and 3 (who did not receive training) may indicate that KMC without any explicit communication-interaction training may also have a positive effect on the participants’ knowledge, mother-infant communication interaction and types of behaviours. The finding is supported by numerous studies indicating the positive effect of KMC on mother-infant communication interaction during the neonatal period and beyond.^1, 21^

It appears that the formal neonatal communication-intervention training made a difference to the participants’ knowledge of their infants. The lack of knowledge amongst all participants (Group 1 without training, Groups 2 and 3) points to a crisis of information about their children's development, as stated by Guralnick^22^ and experienced by mothers.

The results are interpreted with caution, since a small sample of 30 participants was utilised. It was also not possible to randomly allocate the participants to different groups, as trainee participants could not be removed from those who were untrained. The 20-bed KMC unit where the research was conducted is in the form of a dormitory, with mothers continuously in close contact with one another. Therefore, the participants who were untrained would have observed the training and could have copied behaviours seen in the trained participants. The results indicate only the short-term effects of the interdisciplinary neonatal communication-intervention programme on the mothers and not their infants’ development, and no long-term outcomes can be deduced.

Confounding factors could include that the groups were not matched for the infants’ ages, or the number of days in KMC or in the neonatal unit. The participants in Group 1 knew the researcher well due to the three training sessions, and the familiarity with the context could have given this group an unfair advantage over the other groups who only met the researcher once. The results could have been more robust if a fourth group of participants had been included in the hospital that did not routinely practice KMC. The fourth group should have received training without conducting KMC, thereby isolating the impact of the training programme. As only mothers who could communicate well in English were selected for the study, the transferability of the results is limited.

The positive results of Group 1 with training may indicate that the neonatal communication-interaction programme adds value to the evidence-based practice of KMC in the hospital where the training was conducted, and should become part of the routine KMC programme. The challenge ahead is to implement the training programme on a sustainable basis. Further research should determine the long-term effects of the training programme and determine the effects on both the mother-infant communication interaction and infant auditory, speech and language development.
